# From *biocolonialism* to emancipation: considerations on ethical and culturally respectful omics research with indigenous Australians

**DOI:** 10.1007/s11019-023-10151-1

**Published:** 2023-05-12

**Authors:** Gustavo H. Soares, Joanne Hedges, Sneha Sethi, Brianna Poirier, Lisa Jamieson

**Affiliations:** Australian Research Centre for Population Oral Health, The University of Adelaide, Adelaide, SA Australia

**Keywords:** Indigenous peoples, Bioethics, Aboriginal and Torres Strait Islander Peoples, Genetics, Genomics, Indigenous health

## Abstract

As part of a (bio)colonial project, the biological information of Indigenous Peoples has historically been under scientific scrutiny, with very limited benefits for communities and donors. Negative past experiences have contributed to further exclude Indigenous communities from novel developments in the field of omics research. Over the past decade, new guidelines, reflections, and projects of genetic research with Indigenous Peoples have flourished in Australia, providing opportunities to move the field into a place of respect and ethical relationships. This review explores the ethical and cultural implications of the use of biological samples from Indigenous communities in biomedical research. A structured framework outlining emerging topics of interest for the development of respectful omics research partnerships with Indigenous Australians is presented. This paper highlights aspects related to Indigenous governance, community and individual consent, respectful handling of biological samples, data management, and communication in order to protect Indigenous interests and rights and to promote communities’ autonomy.

## Introduction

Omics research (an emerging field of study that encompasses genomics, proteomics, epigenomics, and metabolomics) has provided state-of-art medical technologies to diagnose rare conditions, indicate prognoses, and inform targeted therapies for cancer (Karczewski and Snyder 2018). Despite the relevant contributions to improving health outcomes, advances in this field seem to benefit primarily privileged groups in society. Researchers have voiced concerns regarding the lack of diversity in omics studies and its potential for widening existing health and healthcare inequities (Baynam et al. 2017; Popejoy and Fullerton 2016). Genetic studies worldwide have been heavily dominated by populations of European ancestry (83.2%), whereas Indigenous Peoples contribute to only 0.02% of all samples (Mills and Rahal 2019). Similarly, the representation of Aboriginal and Torres Strait Islander (hereafter respectfully referred to as Indigenous) participants in Australian biobanks is markedly low, ranging from 0 to 1% (Elsum et al. 2019).

Higher participation of Indigenous Peoples in omics studies is essential to obtaining population genetic variation data and promoting equal access to genomic technologies across disadvantaged populations (Mills and Rahal 2019). In the context of Indigenous Australians, the lack of adequate participation in omics research coupled with rapidly advancing medical technology represents a potential hindrance to national efforts of mitigating existing health inequalities (Elsum et al. 2019). The PROPHECY Diabetes Multi-omics Cohort Study is a project led by the South Australian Health and Medical Research Institute (SAHMRI) with a large sample of Indigenous Australians. It illustrates how omics research can be used to improve our understanding of the burden of highly prevalent diseases among Indigenous communities while acknowledging the role of social, political, cultural, and environmental factors in the determination of the disease (SAMHRI, 2022).

In Australia, non-Indigenous men and women live, on average, 8.6 and 7.8 years longer than Indigenous men and women, respectively (Australian Institute of Health and Welfare 2020). Chronic diseases account for approximately 80% of the observed life expectancy gap, whereas another 10.6% of the gap is attributed to deaths due to specific forms of cancer (cancer of the respiratory and intrathoracic organs, cancer of the digestive organs, and cancer of the oropharynx) (Australian Institute of Health and Welfare 2011). Yet, genomic studies examining associations with conditions that largely burden Indigenous Australian communities such as diabetes (Busfield et al. 2002), chronic kidney disease (Thomson et al. 2019), rheumatic heart disease (Gray et al. 2017), and cancer are largely under-explored (McWhirter et al. 2014).

Biological research plays a key role in improving the health outcomes of Indigenous communities and, ultimately, contributing to closing the gap between Indigenous and non-Indigenous Australians (Baynam et al. 2017). Indigenous populations may benefit from omics studies by obtaining relevant information on risk to disease, particularly regarding rare heritable conditions that affect their communities, more accurate prognostic models compared to standard methods, and the development of targeted therapies (Kowal 2012). Promoting an equitable integration of omics technologies into healthcare can facilitate early diagnosis and improve the overall patient journey of Indigenous Australians (Dalach et al. 2021).

Despite the promising advances for the health of minority groups, omics studies still have the potential to both benefit and harm Indigenous communities. Troubling research practices (e.g., lack of adequate consent processes, stigmatisation, exploitation of biological samples and traditional knowledge, lack of direct benefits for communities, and disregard for cultural systems and communities’ priorities) have resulted in controversial genetic studies being conducted with Indigenous Peoples from several countries, including Australia, New Zealand, Canada, Brazil, and United States (Dodson and Williamson 1999; Garrison et al. 2019; Guedes and Guimarães 2020; Perbal 2013). These negative and unethical experiences often led to enduring community harm that have not yet been satisfactorily addressed by the academy (Guedes and Guimarães 2020). Lack of tangible benefits, failure to address community health priorities, disregard for cultural and spiritual traditions, misuse of biological samples, and stigmatisation are some of the main concerns raised by Indigenous Peoples regarding unethical behaviour in omics studies (Garrison et al. 2019; Kowal et al. 2012; McWhirter et al. 2012).

The imperative of observing the highest ethical standards of research not only protects Indigenous communities from potential harm, but can also enable a more active participation in omics studies that are relevant and culturally safe for Indigenous groups (Cheng et al. 2021; Kaladharan et al. 2021; Tong et al. 2020). This study presents a review of the ethical and cultural implications of the use of biological samples obtained from Indigenous communities in biomedical research. Based on relevant research experiences, we provide a structured framework for co-designing culturally appropriate protocols for the respectful management of biological samples in the context of Indigenous health research in Australia.

As researchers working in the field of Indigenous health, we would like to acknowledge how our intersecting identities, experiences, and epistemological standpoints influence our research. GHS, BP, and SS are non-Indigenous early-career researchers and, in the context of Australia, immigrants born on different continents. JH is a Yamatji woman from the mid north-western region of Western Australia who has been working in Indigenous oral health as a senior researcher for over a decade. LJ is a female scholar born in New Zealand who is recognised as a leading researcher in Indigenous oral health. By conducting research committed to reducing health inequalities between Indigenous and non-Indigenous populations, we have all had extensive experience working with distinct Indigenous communities over the years. Our work has been influenced by the recognition of social, political, cultural, and commercial determinants of health, the effects of neoliberalism on health, and decolonial research.

## Dismantling biocolonialism

The scientific (mis)use of Indigenous People’s biological information is inscribed in the historical context of colonialism. The theft of Indigenous remains such as bones, hair, and blood for the purpose of displaying in museums and medical schools overseas was a common practice of colonial settlers in Australia between the late 18th and the early 20th centuries (Turnbull 2007). The scientific discourse prevailing at the time gave rise to erroneous and harmful theories of the evolutionary incapacity of Indigenous Peoples to adapt to the European culture. These pernicious notions of racial difference served as the rationale for several oppressive policies created by the colonial state to legitimise the dispossession of Indigenous Peoples, with enduring effects to their societies and cultures (Turnbull 2022).

In the context of modern science, the most controversial omics studies with Indigenous Peoples fall within the scope of population genetics, a field that investigates the genetic composition of populations and the changes resulting from evolutionary processes. The Human Genome Diversity Project (HDGP 1991–1997) was an emblematic case, being labelled by Indigenous leaders in Australia as “the vampire project.” Indigenous Peoples were portrayed in the HDGP as “Isolates of Historical Interest” whose genetic information should be collected before “vanishing” (Dodson and Williamson 1999). The project was met with strong opposition by Indigenous Australians, organisations, and groups of scientists, raising important discussions around the exploitation of Indigenous genetic material for commercial purposes, biopiracy, lack of consultation process and consent mechanisms, immortalisation of Indigenous cells, indiscriminate use of biological samples, and the impact of genetic studies on Indigenous communities (Dodson and Williamson 1999).

The history of flawed research practices regarding Indigenous biological materials constitutes an expression of *biocolonialism*, that is, a relationship of scientific dominance towards the objectification of Indigenous Peoples’ biological information (Barker 2019). For centuries, biological information of Indigenous groups and their ancestors has served scientists to answer a range of evolutionary questions, with very limited benefits for communities (Kowal 2013). Unsurprisingly, the past scientific experiences deeply rooted in the exploitation of Indigenous bodies persist as one of the main barriers for meaningful collaborations with Indigenous communities in the field of omics research (Kowal et al. 2012; Kowal 2012).

Nonetheless, as described by Kowal, the widespread notion that Indigenous Peoples are not interested in genetics studies has been gradually debunked in Australia (Kowal 2012). Since 2010, as a result of a comprehensive process of consultation with Indigenous communities across the country, Australia’s National Health and Medical Research Council has listed genetics as one of the strategic priorities for Indigenous health research (Kowal et al. 2011). The provision of funding for genetic studies with Indigenous Australians has allowed the development of important partnerships between communities and researchers. For instance, a study investigating the occurrence of vulvar cancer among a group of Indigenous women from the Northern Territory developed mechanisms for community consultation, Indigenous control of the research, and community consent (McWhirter et al. 2014). Epigenetics has been particularly embraced by Indigenous Australians as a culturally relevant approach to examining the intergenerational effects of historical trauma on biological mechanisms (Warin et al. 2019).

Other remarkable examples of mechanisms implemented to engage and consult with Indigenous communities for the purpose of omics studies have emerged across different organisations in Australia. The National Centre for Indigenous Genomics (NCIG) at the Australian National University and the Aboriginal Heritage Project at the University of Adelaide created institutional policies to include Indigenous communities in the governance of their biological collections (Australian National University; University of Adelaide). Furthermore, important guidelines for omics research with Indigenous populations have been developed by research institutions, covering concerns of Indigenous communities that are not sufficiently addressed by national guidelines on research ethics (Kowal et al. 2011; QMIR Berghofer 2019; South Australian Health and Medical Research Institute 2014).

Contemporary initiatives of omics research with Indigenous populations face the challenge of addressing the wider social, political, and cultural contexts in which communities and researchers stand. Omics research must recognise the multiple ways in which broader social determinants influence the health of Indigenous populations, preventing discourses of genetic determinism that reduce the complex disease burden experienced by Indigenous Australians to biological mechanisms (McWhirter et al. 2012). Reflecting on personal trajectories, values, and implicit biases provides a particularly important opportunity for researchers to identify the multifaceted ways in which their positions affect the research process and may play a role in upholding unequal power dynamics (Mc Cartney et al. 2022). It has become a common and well-accepted practice for researchers working in Indigenous health to disclose their personal standpoints through positionality statements in the text of academic publications (Poirier et al. 2022). It provides an opportunity for researchers to critically reflect on how individual experiences and principles influence the ways in which research is conducted (Reid 2020).

Promoting more ethical and meaningful collaborations in the arena of omics research with Indigenous populations requires consistent efforts toward emancipatory practices. The acknowledgement and recognition of Indigenous sovereignty and autonomy is a guiding principle for protecting Indigenous communities’ interests and enabling community control throughout the research process (Garrison et al. 2019). Under an emancipatory paradigm, omics research must address communities’ health priorities, and the knowledge generated should serve to benefit Indigenous donors, their descendants, and their communities (Dalach et al. 2021; Kowal 2012). Increasing emphasis has been placed on the importance of embedding the research process with Indigenous values, collaborating with communities to develop ethical, and culturally respectful protocols, and recognising the contributions of lived experiences from community members (Cheng et al. 2021; McWhirter et al. 2012; Tong et al. 2020). These positive experiences have the potential to move the landscape of omics research with Indigenous Peoples into a place of reflection, respect, self-determination, and ethical relationships.

Based on a growing body of literature reporting omics research collaborations with Indigenous communities in Australia, we identified five main categories of considerations for ethical and culturally respectful research practices: (1) governance, (2) consent, (3) respectful handling of biological materials, (4) data management, and (5) communication. This review does not provide an exhaustive list of the ethical implications for biological research with Indigenous Peoples but presents rather an overview of emerging topics of interest for the development of respectful and culturally-safe omics research partnerships with Indigenous Australians (Table 1). A critical assessment of the topics discussed in this paper might be helpful to inform researchers working in partnership with Indigenous Peoples from other contexts. The set of ethical and culturally respectful practices for omics projects with Indigenous Australians comprise a work in progress that should be further discussed collaboratively with communities.

**Table 1:** Framework of emerging issues for co-designing culturally appropriate omics research protocols with Indigenous Australians

Governance	1. Model	Identify the preferred model of governance and input within each community.
	2. Roles	Outline the roles of the Indigenous governance body.
	3. Issues	Define mechanisms for addressing unanticipated issues related to the project as they arise.
**Consent**	1. Representatives	Identify community representatives who can speak on behalf of collectives and provide community consent.
	2. Community consent	Identify the processes through which community consent should be obtained.
	3. Dynamic consent	Develop mechanisms for a dynamic consent model (providing opportunities at different stages of the research for the participants to withdraw their consent, “renew” their consent, or grant consent for further use of their data) according to community preferences and expectations.
	4. Information	Outline the information that should be provided for participants before initiating the collection of blood samples (identify the questions regarding the handling of the blood samples that participants would want answered).
	5. Delivery	Identify communities’ preferences regarding how information should be delivered, presented, and recorded during the consent process.
	6. Disposal and repatriation	Identify whether preferences related to repatriation and disposal of blood samples should be record as part of the individual consent process or as a community agreement.
	7. Concerns, risks, and benefits	Explore the communities’ worries and concerns regarding the potential uses of biological samples, potential harms, and perceived benefits of the research.
	8. Individual results	Develop mechanisms for recording participants’ preferences regarding obtaining individual tests results.
	9. Rights of non-participants	Develop mechanisms for protecting the rights of family and community members who did not provide consent but share genetic information with participants regarding privacy and access to information.
**Respectful handling of biological samples**	1. Ownership	Explore the communities’ understandings regarding the ownership of biological materials.
	2. Meaning	Explore the communities’ understandings regarding the cultural and spiritual meanings of biological samples.
	3. Collection of samples	Explore the communities’ preferences regarding how samples should be collected, by which professionals, and in which settings.
	4. Handling and storage	Develop protocols for safe and respectful handling and storage of biological samples throughout all stages of the research
	5. Secondary uses	Identify in which circumstances, if any, it is acceptable to use samples for secondary purposes or further research.
	6. Withdraw	Develop mechanisms for withdrawing samples at request of participants or communities.
	7. Return of samples	Develop culturally sensitive protocols for the repatriation of samples if requested by communities or participants, with expected timeframes.
	8. Disposal	Develop mechanisms for continuous community advice regarding the appropriate disposal of blood samples.
	10. Participants who passed	Develop protocols for consulting family members regarding the use, repatriation, or disposal of blood samples from participants who passed away.
**Data management**	1. Custody	Define how the data should be stored, who will be the data custodian(s), and who might have access to the data.
	2. Access	Define whether access to data and/or samples might be considered in the future and which protocols should be followed to grant access.
	3. Community	Outline how the data should be made available and accessible for communities and participants, and at which level (individual, community, or whole population).
**Communication**	1. Communication strategy	Build a communication strategy that outlines the preferred methods of reporting the study findings to communities, the frequency and expected timeframes.
	2. Community input	Define mechanisms for incorporating community input on study reports.
	3. Knowledge translation	Develop a knowledge translation plan that considers the community expectations regarding the translation of research findings to practice, policy, and public dissemination.

### Indigenous governance

Enabling Indigenous control of the research process is central for addressing the power imbalance in research relationships between communities and non-Indigenous scholars (Pratt et al. 2022). Aligned with the principle of sovereignty, Indigenous governance allows communities to set the terms on how they want to be involved in omics projects while honouring Indigenous cultural and social values. Researchers, in collaboration with Indigenous Peoples, have developed a range of mechanisms for community governance of omics initiatives, with varying levels of involvement and input. Tong et al. described a governance structure for a genomic-wide association study of rheumatic heart disease with Indigenous Australians that included three subdivisions: a clinical, a scientific, and an Indigenous governance committees (Tong et al. 2020). The role of the Indigenous committee, formed by three Indigenous chief investigators, included involvement in the design of the study protocol, the right to reject protocol modifications, and provision of guidance to the project team. In a study investigating vulvar cancer among young Indigenous women, McWhirther et al. (2012) conducted extensive consultations with community members and organisations. An Indigenous Reference Group comprising women from all the communities included in the study was created to guide the development of the research and facilitate the communication of genetic information to the local population.

Recently, researchers proposed a protocol for the involvement of Indigenous communities in the design of genomic research (Cheng et al. 2021). Indigenous governance of the project will be achieved through an equal ratio of Indigenous and non-Indigenous chief-investigators, in addition to Project Advisory Committees chaired by Indigenous elders nominated by communities. The project will include workshops with community members to co-design strategies related to the recruitment of participants, consent, collection and storage of DNA, data sovereignty, and the dissemination of research findings. Committees of Indigenous representatives have also played an important role in assessing research applications and granting approval for the use of data or blood samples from Indigenous donors stored in biobanks (Australian National University; Cunningham and Dunbar 2007).

### Consent

Developing appropriate mechanisms for obtaining community and individual consent is a critical step in omics partnerships with Indigenous communities. Dodson and Williamsom (1999) argue that obtaining biological samples from Indigenous participants without prior group consent might be regarded as an invalid and unethical practice. The use of Indigenous biospecimens in omics research intersects with the diversity of cultural meanings that blood and genetic information hold for Indigenous Australians, including for aspects related to group identity, community ties, and spirituality (Kowal et al. 2015). Community consent acknowledges the collective ownership of Indigenous biological materials given that genomic information is often shared by community members. It also protects community interests and the rights of members who are not directly involved in the study but whose genomic information might be used for scientific purposes (McWhirter et al. 2012). Different formats of group and individual consent have been discussed in the context of Indigenous omics research. For instance, broad consent has been deemed insufficient to protect against community harm such as the publication of stigmatising research findings. Broad or ‘blank’ consent, that is, when participants are asked to contribute to collections of biological specimens without clear and specific research aims, is by definition uninformed (Kowal 2015).

Given the complex and often prolonged nature of omics projects, it is expected that researchers provide recurring opportunities for Indigenous participants and communities to renew, renegotiate, or withdraw consent (Sharp and Foster 2002). Dynamic consent, an approach that has been increasingly adopted in the field of biomedical research, provides a mechanism for sustained communication between participants and the research team during all stages of the research (Prictor et al. 2018; Teare et al. 2021). Although dynamic consent is generally established using digital platforms, its philosophical underpinnings could be extended to regular face-to-face meetings organised to discuss the concerns of the Indigenous communities and to allow participants to revisit the records of their decisions (Prictor et al. 2020).

Researchers must meet communities’ expectations regarding the delivery, clarity, and type of information provided during consent procedures. Indigenous participants recruited for omics studies in Australia have expressed preferences for the use of visual aids, culturally appropriate concepts, and exclusion of medical terminology and acronyms (Davies et al. 2014). Participants have often expressed the desire to obtain pertinent information regarding how samples are stored and managed, including the type of container used, how they will be preserved, the security of the facilities, and when they will be disposed (Hiratsuka et al. 2012). Other relevant topics to be disclosed include how data will be protected and managed, and when the community should expect reports on the research progress and the return of findings (Arbour and Cook 2006). Mechanisms might be included in the consent procedures for recording the participants’ preferences regarding obtaining individual and potentially sensitive findings, secondary use of biospecimens, long-term storage, and disposal and repatriation of samples (Sharp and Foster 2002).

Considering that notions of risks and benefits are culturally shaped, researchers must take into account the perspectives and lived experiences of community members when discussing the risks and benefits of the project (Kowal 2015). Simply de-identifying the data from personal information is not sufficient to mitigate potential community harm related to the publication of stereotyping findings that may lead to increased discrimination against Indigenous Peoples (Rothstein 2010). A clear plan for monitoring and mitigating risks for participants and communities should be explicitly stated and put in place, while tangible benefits should be offered by the project (for example, offering training and positions for community members as research officers) (Kowal et al. 2015).

### Respectful handling of biological samples

The recognition of Indigenous perspectives regarding the cultural meanings attributed to biological materials is critical for the development of appropriate protocols for omics studies (Kowal et al. 2015). Given the immense sociocultural diversity of Indigenous Peoples, researchers should not assume homogeneous systems of attitudes and beliefs across communities. Proper consultation with the groups involved in the study can ensure that procedures related to the collection, storage, use, destruction, and repatriation of biological samples are conducted in accordance with the local cultural values and expectations (QMIR Berghofer 2019). As samples obtained from Indigenous individuals may hold important cultural and spiritual meanings, the same respectful treatment conferred to communities and participants should be extended to the management of the biological samples (Arbour and Cook 2006). All members of the research team should acknowledge the significance and value of the samples to their donors and communities (QMIR Berghofer 2019).

Biospecimens can provide information that is perceived as relevant for both Western and Indigenous epistemologies and healing systems (Kowal 2015). Given the powerful connections between biological tissues, ancestors, culture, and land, water, and skies, it is strongly recommended that biological materials from Indigenous communities be stored in country (that is, samples should be securely kept in facilities located within national boundaries or, where appropriate, close to traditional Indigenous lands) (Kowal 2015). The locations where samples and the derived data will be stored and processed should be clearly discussed with communities, as Indigenous belief systems might prevent removing Indigenous biological materials to laboratories overseas (QMIR Berghofer 2019). Similarly, the principle of connection to land has implications for the repatriation of samples. Indigenous communities should be given the opportunity to decide under which circumstances they require the return of their biological materials to traditional lands. Clear mechanisms should be developed to determine which materials should be returned (e.g., saliva, blood, genomic data), the culturally-appropriate processes for disposal or repatriation of samples, the expected timeframes, and how individual or collective preferences will be recorded (QMIR Berghofer 2019).

Co-designing research protocols with the Indigenous communities is encouraged to establish culturally-appropriate procedures for the use and storage of the biological samples. Reflecting the process of consultations and negotiations with communities, protocols should explicitly outline the methods, facilities, and timeframes adopted for the storage of the samples, who will be granted access to the collections, and which procedures will be put in place for disposing or repatriating the biospecimens (QMIR Berghofer 2019; South Australian Health and Medical Research Institute 2014). Moreover, there is a general movement proposing that the ownership of all biological materials obtained from Indigenous populations in the context of research remains with the participants and communities involved (Arbour and Cook 2006). While the samples would be treated as the unalienable property of Indigenous donors, the research team would be considered the guardian of the biospecimens “loaned” for the purposes of the research. Indigenous participants confer the stewardship of their biospecimens to the research team based on a relationship of trust and relationality. Honouring the good faith of the research participants who contribute to the study with their own biological information, researchers are compelled to discuss with communities whether scientists not directly involved in the study or commercial laboratories should be granted access to samples at any stage of the project (QMIR Berghofer 2019).

### Data management

Open science and Indigenous data sovereignty are two distinct and flourishing movements in modern research that pose relevant implications for omics studies with Indigenous Peoples – often in conflicting directions. While genomic research has consistently adhered to principles of open science and unrestricted sharing of data, concerns have been raised about its potentially damaging and inequitable outcomes for Indigenous populations. Mc Cartney and colleagues argue that the fully open model of sharing genomics data must be questioned in the context of Indigenous health research (Mc Cartney et al. 2022). Open science fails to protect Indigenous interests such as the respectful communication of findings and the sharing of research benefits with communities. The lack of Indigenous control over the management and uses of their biological information reinforces power imbalances and colonial values, which could ultimately increase the exclusion of vulnerable populations from omics research (Hudson et al. 2020; Mc Cartney et al. 2022).

Flexible approaches for the management of omics data should be developed in recognition of the history, interests, and perspectives of the communities involved in the study. Indigenous data sovereignty promotes the right of Indigenous communities and participants to exercise control over all the processes related to data, including management, stewardship, analysis, interpretation, dissemination, and reuse (Carroll et al. 2021). Data governance structures centred on Indigenous self-determination and accountability that empower Indigenous communities to meet their aspirations are needed. In Australia, the Maiam nayri Wingara Indigenous Data Sovereignty Collective has explicit recommendations for promoting a data ecosystem aligned with the principle of Indigenous data sovereignty such as reporting data at the individual, community, and Indigenous Nation levels (Maiam nayri Wingara Indigneous Data Sovereignty Collective). Moreover, Indigenous communities retain the right to express how and through which processes they would like to exercise control over the sets of omics data. A thorough and transparent data management plan should be developed in collaboration with communities involved in the study addressing issues related to the custody of data, whether access to records will be considered over time, and how Indigenous control can be established (QMIR Berghofer 2019).

### Communication

Regularly reporting findings to communities might provide opportunities for maintaining levels of engagement and trust, incorporating the voices of participants into publications, and portraying discoveries in culturally sensitive ways that take into account community values and concerns (Arbour and Cook 2006; Sharp and Foster 2002). A clear communication strategy should be discussed and co-developed with communities, addressing aspects related to the dissemination of findings, knowledge translation, and reporting of research outcomes to stakeholders and participants. Allowing communities to review study findings before public release is perceived to substantially reduce the risks of publishing findings that may lead to genetic stereotyping and discrimination towards Indigenous groups (Cormack et al. 2019; Sharp and Foster 2002). Research findings should be returned to communities in a way that promotes their autonomy, advocacy, and self-determination. Indigenous Peoples should not only be able to use omics information independently to support their own development, but also in collaboration with researchers. A plan for knowledge translation that supports the integration of omics findings into practice and policy development is essential to enable participants and communities to access research benefits (QMIR Berghofer 2019).

## Discussion

A flourishing body of literature reporting relevant omics collaborations with Indigenous Australian communities has emerged since 2010, when the main statutory authority for medical research included genetic research as one of the strategic priorities for Indigenous health (Cheng et al. 2021; Kaladharan et al. 2021; Kowal 2012; McWhirter et al. 2012, 2014). The allocation of funding for omics studies with Indigenous Australians has been an important step towards promoting equitable outcomes related to accessing knowledge and technologies. Researchers have developed meaningful approaches for engaging, consulting, and collaborating with Indigenous groups in Australia, providing guiding reflections on the importance of culturally appropriate methods for studies involving biological samples from Indigenous populations (Cheng et al. 2021; Kaladharan et al. 2021; McWhirter et al. 2012).

The movement for greater Indigenous involvement and control in omics studies is aligned with a broader perspective that advocates for participatory research and decolonial methods in Indigenous health research (Braun et al. 2014; Kite and Davy 2015; Sharmil et al. 2021). Several issues identified in this review such as Indigenous data governance and community consent are relevant for research across multiple health disciplines, but present special implications for omics studies given the particularities and sensitivity of the field for Indigenous communities (Mc Cartney et al. 2022; McWhirter et al. 2012). Applying principles of decolonial research to studies that require the manipulation of Indigenous biological information has the potential to challenge the established practices that prevent Indigenous communities from benefiting from omics research.

It is important to bear in mind that recommended practices and guidelines should be considered in the context of Indigenous communities rather than Indigenous individuals. In other words, this discussion is relevant for studies that recruit participants based on their kinship or ties to an Indigenous group. Studies that eventually recruit Indigenous participants but do not directly engage with an Indigenous community might not benefit from the principles here discussed. We also acknowledge that the proposed research practices might not be applicable to several contexts (for example, Indigenous participants living in urban settings).

*Indigenous* is a generic term used to signal a rich diversity of Traditional Peoples with unique identities, cultures, languages, and beliefs. Indigenous Peoples share common characteristics such as a historical continuity with pre-settler societies, strong ties to traditional lands, holistic views on health and wellbeing, and distinct cultural, political, and economic systems to that of dominant societies (United Nations). We recognise that these groups are not homogeneous and, as such, we present principles that can be incorporated and adjusted to local realities. Furthermore, considering the large-scale of omics projects, researchers are likely to work alongside multiple Indigenous communities, demanding adjustments to local characteristics and needs.

Indigenous sovereignty is the key guiding principle for the development of omics research that respects communities’ autonomy, interests, and values. It not only places emphasis on community self-determination throughout the research process, but also recognises the diversity of Indigenous groups and their multiple worldviews (Garrison et al. 2019). A single model with prescriptive guidelines for omics research would not adequately respond to the heterogeneity of Indigenous communities and cultures (Kowal et al. 2015). In acknowledging Indigenous sovereignty, researchers and communities might establish culturally-safe approaches for the study, addressing local concerns and cultural characteristics.

Recent research experiences describing meaningful mechanisms of negotiation, consultation, and collaboration with Indigenous communities can set the foundations for a future of equitable and culturally appropriate practices in omics research with Indigenous Peoples, overcoming a legacy of exploitation and violations. It is important to bear in mind, however, that transitioning towards an emancipatory research paradigm that is centred on Indigenous values and self-determination is not a steady and linear process. The elements identified in this review cannot entirely address the ethical, anthropological, and philosophical dilemmas in omics research with Indigenous populations as different issues are likely to emerge with the development of new omics technologies (Ilkilic and Paul 2009). Important questions remain to be answered such as how to identify the individuals with authority to speak on behalf of a given community and a community’s preferred model of governance. Furthermore, researchers must commit to navigating the opportunity to shift the control over the research course to the hands of Indigenous communities in order to promote changes in the culture of omics research (Duke et al. 2021). Given the magnitude and complexity of such enterprise, research involving the use of Indigenous biological information can only serve Indigenous interests if their voices and leadership are properly valued throughout the research process.

## Conclusion

The movement for greater Indigenous involvement and control in omics studies aligns with a broader perspective advocating for participatory research and decolonial methods in Indigenous health research. Implementing these principles requires acknowledging the diversity of Indigenous communities and their multiple worldviews, as well as committing to shift control over the research course to the hands of Indigenous communities. While there are still ethical, anthropological, and philosophical dilemmas to be addressed, recent research experiences describing meaningful mechanisms of negotiation, consultation, and collaboration with Indigenous communities can set the foundations for a future of equitable and culturally appropriate practices in omics research with Indigenous Peoples.
